# Acute Respiratory Diseases and Carboxyhemoglobin Status in School Children of Quito, Ecuador

**DOI:** 10.1289/ehp.7494

**Published:** 2005-01-14

**Authors:** Bertha Estrella, Ramiro Estrella, Jorge Oviedo, Ximena Narváez, María T. Reyes, Miguel Gutiérrez, Elena N. Naumova

**Affiliations:** ^1^Corporación Ecuatoriana de Biotecnología, Quito, Ecuador;; ^2^Universidad Central del Ecuador, Quito, Ecuador;; ^3^Baca-Ortiz Children Hospital, Quito, Ecuador;; ^4^Fundación Natura, Quito, Ecuador;; ^5^Tufts University School of Medicine, Boston, Massachusetts, USA

**Keywords:** acute respiratory infections, carbon monoxide exposure, carboxyhemoglobin, children, Ecuador, traffic-related pollution

## Abstract

Outdoor carbon monoxide comes mainly from vehicular emissions, and high concentrations occur in areas with heavy traffic congestion. CO binds to hemoglobin, forming carboxyhemoglobin (COHb), and reduces oxygen delivery. We investigated the link between the adverse effects of CO on the respiratory system using COHb as a marker for chronic CO exposure. We examined the relationship between acute respiratory infections (ARIs) and COHb concentrations in school-age children living in urban and suburban areas of Quito, Ecuador. We selected three schools located in areas with different traffic intensities and enrolled 960 children. To adjust for potential confounders we conducted a detailed survey. In a random subsample of 295 children, we determined that average COHb concentrations were significantly higher in children attending schools in areas with high and moderate traffic, compared with the low-traffic area. The percentage of children with COHb concentrations above the safe level of 2.5% were 1, 43, and 92% in low-, moderate-, and high-traffic areas, respectively. Children with COHb above the safe level are 3.25 [95% confidence interval (CI), 1.65–6.38] times more likely to have ARI than children with COHb < 2.5%. Furthermore, with each percent increase in COHb above the safety level, children are 1.15 (95% CI, 1.03–1.28) times more likely to have an additional case of ARI. Our findings provide strong evidence of the relation between CO exposure and susceptibility to respiratory infections.

Numerous studies have found a strong association between respiratory illness and exposure to traffic-related air pollution (Choudhury et al. 1997; Hajat et al. 2002; Polosa et al. 2002; Romieu et al. 2002; Shamsiiarov et al. 2002; Spinaci et al. 1985). Traffic-related nitrogen monoxide, nitrogen dioxide, black fumes, and ammonia particulate have been linked to an increase in respiratory symptoms and a decrease in pulmonary function in school-age children (Boussin et al. 1990; Keiding et al. 1995; Lercher et al. 1995; Lwebuga-Mukasa et al. 2003; Quian et al. 2000; Steerenberg et al. 2001; van Vliet et al. 1997; Wjst et al. 1993; Yang et al. 1998, 2002). Carbon monoxide, a toxic product of incomplete combustion, can also impair respiratory function. High CO concentrations may occur in areas with heavy traffic congestion, especially in urban settings with insufficient emission regulation. The main indoor sources of environmental CO are smoking and domestic fuel combustion with inadequate stoves and furnace ventilation (Collings et al. 1990; Kleinman 2000; Puente-Maestu et al. 1998). Although the physiology and adverse effects of acute CO poisoning on the respiratory system are well documented, very few studies have been conducted to understand the effects of chronic low-dose CO exposures on susceptibility to respiratory infections.

Carboxyhemoglobin (COHb), a marker for CO exposure, reflects the binding of CO to the hem portion of hemoglobin capturing oxygen. A concentration of COHb < 2.5% is currently considered safe (Kleinman 2000). The lowest level of COHb, at which adverse effects are observed, ranges from 2.9 to 3% [U.S. Environmental Protection Agency (EPA) 2000]. COHb concentrations of 5–10% serve as an indicator of acute CO poisoning and are associated with impaired visual function, task performance, and maintaining alertness (Raub and Benignus 2002). Even a relatively low CO exposure may increase COHb levels in human peripheral blood (Raub and Benignus 2002). Higher levels of COHb have been observed in smokers compared with nonsmokers (Behera et al. 1991). In addition, children living in households with smokers or wood/coal/gas heating systems exhibit slight increases in COHb levels (Vazquez et al. 1997).

The effects of CO exposure at high altitudes may be more detrimental than exposure at sea level. In the presence of high CO concentrations, a compensatory mechanism of adaptation to low oxygen saturation in high altitude that leads to increased production of red blood cells may be insufficient. High-altitude residents have a greater initial body burden of COHb and may attain the COHb level associated with the U.S. National Ambient Air Quality Standard for CO more quickly than sea-level residents (McGrath 1989). The respiratory effects of chronic exposure to CO in high-altitude populations have not been explored.

The objectives of this pilot study were *a*) to compare the incidence of acute respiratory infections (ARIs) in school-age children living in three communities that differ in traffic intensity in urban and suburban areas of Quito, *b*) to examine the relationship between ARI occurrence and individual COHb concentrations, and *c*) to examine the joint effect of COHb levels (a measure of CO exposure) and hematocrit levels (a measure of a compensatory oxygen-delivery function) on the incidence of ARI. To achieve these goals, we conducted a 12-week prospective study of 960 children attending elementary schools in the early spring of 2000 in Quito, the capital of Ecuador. Quito is a rapidly developing city with > 1 million residents of relatively homogeneous ethnicity. It is located 2,825 m above sea level and enjoys a mild climate year round, but is challenged by heavy air pollution, 82% of which is due to vehicle exhaust. Substantial human morbidity and mortality in Quito are likely linked to environmental factors.

## Materials and Methods

### Study design.

From January through April of 2000, we conducted a prospective study in young children attending Quito’s public elementary schools. First, three schools were selected that were comparable with respect to the type of school building (with concrete walls and roofs and cemented playgrounds) and the number of children per class (~ 45 children, *p* = 0.56) but differed by traffic intensity in surrounding areas. One school was located north of Quito, in the suburban area of Nayon and represents a low-traffic area (LT-school). The second school was located in a moderate-traffic area of Quito (MT-school). The third school was located in a heavy-traffic area in downtown Quito (HT-school). Next, an initial screening was performed in each school to identify a pool of children eligible for the study. During the screening period, detailed information about the study was delivered to the teachers and to the parents of each child. Children with chronic respiratory illnesses and major congenital and/or chest deformities interfering with the respiratory tract were excluded from enrollment. In each school, 320 children, 6–11 years of age (age was confirmed by birth certificate), who had formal written consent freely signed by their parents, were randomly selected and enrolled in the study. Finally, from the total 960 children enrolled 295 were randomly selected to obtain blood measurements.

### Primary outcome, ARI.

During the 12-week study period, each child was visited in the school twice weekly by a pediatrician who examined the child’s respiratory signs and symptoms to determine the presence of upper and lower ARIs. For each child, the number of episodes of upper and lower ARIs observed over the study period was determined, considering a 2-week period to be free of infections. We adapted ARI case definitions proposed by Sempértegui et al. (1999). Upper ARI was defined as the presence of two or more of the following signs/symptoms: cough, nasal secretion, fever > 37.5°C (auxiliary temperature), inflammation of pharynx, and anterior cervical lymphadenitis. Presence of otitis (local pain, aural pus, and eardrum congestion) was also considered as upper ARI. Lower ARI was defined as tachypnea (respiratory rate > 20) and/or lower respiratory tract secretions (alveolar or bronchoalveolar) assessed by thoracic auscultation, with one or more of the following: fever, cough, and chest retractions.

### Anthropometric measurements.

On the first day of the study, weight and height for all enrolled children was measured by standard procedures using the instruments calibrated by the Ecuadorian Institute of Normalization (Quito, Ecuador). Weight was measured with a DETECTO balance (DETECTO, Webb City, Missouri, USA) and recorded to the nearest 0.1 kg. Height was obtained with a calibrated scale using a fiberglass tape measure and recorded in centimeters. Weight-forage *Z*-score (WAZ), height-for-age *Z*-score (HAZ), and body mass index (BMI) values were calculated.

### Survey.

Baseline measurements for confounders, including household heating and cooking conditions (the use of kerosene or wood), the presence of smokers, and household crowdedness (number of persons/number of rooms), were collected via household surveys. On the first week of the study, a survey was sent to the parents of each child. After 2 weeks, 715 surveys (77%) were returned.

### Blood measurements.

COHb and hematocrit levels were measured on the first day of the study. Venous blood was drawn with plastic syringes and placed into EDTA-treated tubes. Blood was immediately transported for analysis. COHb was measured by spectrometry and expressed as a percentage of plasma hemoglobin. Hematocrit was obtained by centrifuging whole blood in microtubes and expressed as a percentage.

### Statistical analysis.

Data entry and management were performed using Epi-Info 6.04c software (CDC, Atlanta, Georgia, USA). SPSS 11.5 (Lead Technologies Inc. SPSS Inc., Chicago, Illinois, USA) and S-plus 6.0 (Insightful Inc., Seattle, Washington, USA) were used for statistical analysis.

For each child, we estimated the number of ARI episodes observed during the study period and the number of weeks a child had attended the school. The primary health outcome—the annual ARI rate—was expressed as the number of ARI episodes per year per 1,000 children. Descriptive statistics for the primary health outcome, the blood measurements, all baseline measurements, and variables collected via surveys were calculated. Because the blood measurements were not available for all children, we compared descriptive statistics in both subsets, with and without the blood measurements, using *t*-test or test of proportions as appropriate.

To examine the effect of traffic-related pollution on COHb and ARI, we estimated the average COHb concentration and the average rate of ARI for each school and assessed the differences using analysis of variance, hypothesizing that children attending the school located at in the high-traffic area would have the highest level of COHb and the highest incidence of ARI compared with children attending LT- or MT-schools.

To test the hypothesis that children with COHb concentrations above the safe level of 2.5% are more susceptible to ARI, we created two binary variables: one to reflect the occurrence of ARI (0, no ARI; 1, at least one case of ARI), and the second variable to reflect the level of COHb (0, COHb ≤ 2.5%; 1, COHb > 2.5%). A logistic regression model including a set of confounders for adjustment [age, sex, HAZ, WAZ, type of domestic fuel (kerosene or wood), smoking, crowdedness, and hematocrit level] was then applied. Because the household information was not available for all the children in the study, we repeated this model excluding variables on household confounders. The results of modeling were expressed as risk ratios with their confidence intervals (CIs).

To assess the association between the recurrence of ARI episodes and high COHb concentrations, we employed a log-linear Poisson regression model. In this model we predicted the observed number of ARI in a given child by an individual COHb measurement that exceeds the safety level. The model included the same set of confounders as the logistic model. Results were expressed as an adjusted relative risk with its CIs.

To examine interactions between COHb concentration, hematocrit level, and the incidence of ARI, we applied a generalized additive model (GAM) with nonparametric spline smoothing (Hastie and Tibshirani 1990). In this nonlinear model, we regressed the number of cases of ARI against individual levels of COHb and hematocrit. The result of the model was displayed using a three-dimensional surface with x-axes reflecting COHb concentration, y-axes reflecting hematocrit level, and z-axes reflecting the predicted numbers of ARI episodes.

## Results

Of 960 enrolled children, 910 (95%) completed the study (294 in the LT-school, 303 in the MT-school, and 313 in the HT-school). Fifty children were lost in the follow-up because of local migration. A total of 10,729 child-weeks of observation were accumulated in the study (3,382 child-weeks in the LT-school, 3,560 child-weeks in the MT-school, and 3,777 child-weeks in the HT-school). Of 910 children, 715 (78%) completed the household survey. The blood tests were available for a subset of 295 children.

Over the 12-week study period, 848 cases of ARI were detected. Twenty-four percent of the children suffered recurrent ARIs. The overall incidence rate of ARIs was 78.6 cases per 1,000 child-weeks of observation, or 4.05 cases per 1,000 children annually.

We estimated the descriptive statistics for ARI rates, baseline characteristics, survey responses, and COHb and hematocrit measurements for the entire study population and for the two subsets, with and without the blood measurements (Table 1). The ARI incidence and all other measurements, except the percentage of stunted children, did not differ between the two subsets.

## Discussion

The main finding of the study was that COHb concentrations elevated because of traffic pollution correlate with the occurrence of ARIs in young children. A high COHb level was associated with at least one additional case of ARI in a 12-week period or a 3-fold increase in the annual rate of ARI incidence. These associations remain after adjusting for age, sex, weight, height, BMI, hematocrit level, previous history of asthma, presence of smokers, kerosene and/or wood use for cooking, and level of crowdedness. Our findings imply that exposure to a high level of CO, the primary reason for increased COHb concentration, may lead to increased susceptibility to ARIs. Information on chronic CO exposure and the incidence of respiratory disease in a sensitive subpopulation such as children residing in areas with high micronutrient and oxygen deficiency is novel.

The observed COHb level in the studied population was very high. Even the average concentrations exceeded a safe level of 2.5%, mostly due to elevated COHb in children from the area with high traffic volume. Almost half of those children had a COHb level consistent with acute CO poisoning. Unfortunately, routine monitoring for CO in Quito was not conducted at the time of the study. The absence of ambient CO measurements did not allow direct assessment of the relations between COHb concentrations and exposure to CO in the studied population. The sparse CO and particle matter monitoring, performed by the Departmento de Control de la Calidad del Aire (Department of Air Quality Control, Quito) in the central part of Quito in 1995–1999, has demonstrated that the median monthly concentrations of both pollutants consistently exceeded U.S. and European standards (Southgate et al. 1995). Furthermore, in December and January, CO concentrations exceeded the standards three times. It is plausible that the study inception in early January 2000 coincided with the seasonal peak in CO exposure, which contributed to elevated COHb levels.

Indoor CO comes predominantly from smoking and heating/cooking fuel combustion. A gradient of COHb levels in children with different type of heating systems has been observed, 0.88 ± 1.34% for wood and coal heating, 0.58 ± 0.97% for gas heating, and 0.28 ± 0.4% for electric system, although it was not significant (Vazquez et al. 1997). It has been demonstrated that CO inhaled during cooking in high-altitude conditions results in a 1% increase in COHb concentration but does not reach clinically unsafe levels (Keyes et al. 2001). Because of the warm climate of Quito, with air temperature ranging from 10°C (50°F) at night to 25°C (77°F) at noon averaging at 15°C (64°F) year around, the heating systems are not necessary. In our study, we did not observe significant effects of smoking or kerosene and/or wood use for cooking on individual COHb concentrations. Therefore, we concluded that the dominant factor for the significant COHb gradient observed in the study was traffic-related pollution, which was different in the three school locations.

**Figure 1:**
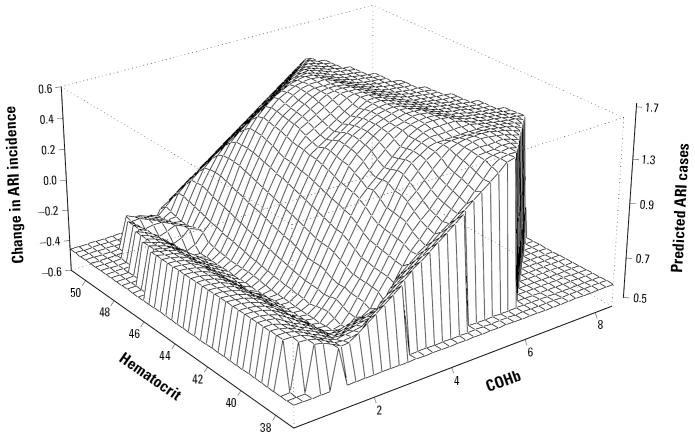
The GAM-predicted joint effect of COHb concentration (x-axes) and hematocrit level (y-axes) on the occurrence of ARIs during the 12-week study period in young school-age children in Quito, Ecuador.

It is important to note that children attending the school located in the central historical district of Quito with the heaviest traffic had the highest levels of COHb and the highest incidence rate of ARIs. Surprisingly, children from the area with low traffic had the lowest COHb levels but did not experience the lowest incidence of ARI. This finding could be explained by the potential influence of other factors that we were not able to consider, such as anemia, immune status, or the presence of local outbreaks of infection. Although the regression analysis demonstrated that the confounders considered were not associated with ARI, it is possible that other factors might influence health outcomes for these suburban children. It is also possible that the slight “ridge” of ARI incidence in Figure 1 at very low COHb levels occurred because the children who had low COHb concentrations (most of them were attending the LT-school) were also more chronically malnourished (stunted) than the children from the two other schools (28 vs. 9 and 8%, respectively) (Table 2). The observed differences in stunting might be indicative of frequent diarrheal diseases and/or malnutrition that occurred in early childhood (Freire et al. 1988). Despite our effort to select schools with similar socioeconomic status, we suspect that the students in the suburban area came from families with lower literacy levels (considering the lowest percentage of completed surveys) and poorer households conditions (considering the high rate of crowdedness and more frequent use of firewood, the cheapest cooking fuel) than the rest of the students. Nevertheless, the subanalysis of potential interactions between stunting and COHb levels in children residing in the low-traffic area revealed that the risk of ARI in children who were stunted and had high COHb level was 3.37 (95% CI, 1.001–11.26), but the sample size was too small to draw a strong conclusion. Therefore, more detailed studies are needed to disentangle the effect of CO exposure from the effect of malnutrition and socioeconomic conditions on ARI.

Although we did not find reports on susceptibility to respiratory infections and elevated COHb, similar associations between COHb level and inflammatory pulmonary diseases has been described (Yasuda et al. 2002). A biologic plausibility for the effect of high CO exposure on bronchial-alveolar system impairing local immune reactivity has been proposed (McGrath 2000); however, direct investigations of CO effects on innate and adaptive immunity are scarce. The following mechanism might explain a local defect of immunoreactivity leading to a high susceptibility to respiratory infection. Given that the mobility of immune cells and the motility of a ciliar epithelium that lines bronchial-alveolar system are highly ATP-dependent processes, the high CO content might directly compete with free radicals for the binding site on cytochrome C during oxidative phosphorylation. This might deprive dendritic cells, B-cells, and T-cells of ATP-dependent cytokine production, immunoglobulin synthesis, and the killing of virus-infected cells (Bona et al. 1999; Caldwell et al. 2001; Loeffler et al. 1990). Considering hemoglobin dependency, the oxygen binding might affect the proliferation of dendritic cells and T-and B-cell reactivity in the lymphoid organs (Shimizu et al. 1996; Taneja et al. 2000). The division of immune cells, primed in bronchial lymph nodes, might be affected because of their high sensitivity to hypoxia even when the inhibitory effect CO on the neural system is not yet detected.

Our pilot study contributes to the body of literature that demonstrates the harmful effect of traffic-related pollution in urban settings. In the last decade, the air quality has been rapidly decreasing in Quito because of urbanization and increasing exhaust from public transportation. According to the Dirección Nacional de Tránsito, Quito, the number of cars registered in Quito rose from 174,875 in 1995 to 209,757 in 1998. Diesel is used in the vast majority of trucks and buses and in 6% of the cars (Jurado 1991). The city lies in a narrow valley with a north–south orientation of encircling mountains and environmental conditions challenged by high volcanic activity and El Niño effects. The polluted air in the city is often stagnant, and its clearance depends mainly on prevailing wind and precipitation. Strict emission regulations, as much as economic development, are crucial to the nation of Ecuador. Unfortunately, economic capacities to address the issues of health and the environment at the municipal and national levels, are severely constrained by harsh economic situations and shifting political factors. Nonetheless, receptivity to environmental concerns is evolving rapidly in Ecuador to one where policy makers are open to these issues.

## Figures and Tables

**Table 1:** The incidence of ARIs and exposure measurements for the entire study population as well for the COHb substudy contrasted with the remaining study participants.

Study parameters	Total (*n* = 910)	COHb Substudy (*n* = 295)	Remaining (*n* = 615)
Measure			
Children with ARI (%)	49.56	50.50	50.40
No. of ARI episodes	848	285	563
Annual rate of ARI	4.05	4.19	3.98
Baseline characteristics (*n*)	910	295	615
Age [years (mean ± SD)]	8.5 ± 1.2	8.4 ± 1.2	8.6 ± 1.2
Females (%)	43.3	40.68	44.6
Weight [kg (mean ± SD)]	26.3 ± 5.8	25.9 ± 1.0	26.4 ± 5.7
Underweight children, WAZ < −2 SD (%)	3.4	4.2	3.1
Height [cm (mean ± SD)]	125.2 ± 9.2	124.6 ± 9.4	125.6 ± 9.1
Stunted, HAZ < −2 SD (%)	16.1	20.7*	13.8
BMI (mean ± SD)	16.6 ± 2.3	16.6 ± 2.2	16.6 ± 2.3
Survey response (*n*)	715	233	482
Completed survey (%)	78.6	78.4	79
Crowdedness (mean ± SD)	1.29 ± 1.1	1.28 ± 0.85	1.29 ± 0.83
Households with kerosene use (%)	2.6	1.8	3.1
Households with firewood use (%)	5.3	5.4	5.2
Smokers (%)	25.5	23.5	26.5
Children with history of asthma (%)	2.5	2.6	2.5
Blood tests (*n*)		295	
COHb [% (mean ± SD)]		2.81 ± 2.19	
COHb > 2.5% (%)		46.4	
Hematocrit [% (mean ± SD)]		43.26 ± 2.6	

The blood tests were performed only for 295 children (our substudy). To avoid redundancy we provided the values only for the substudy. The total will have identical values.

* Significant difference at *p* = 0.02 between groups with and without COHb measurements.

**Table 2:** Incidence of ARIs and exposure measurements for children attending LT-, MT-, and HT-schools.

Study parameters	Low traffic (*n* = 294)	Moderate traffic (*n* = 303)	High traffic (*n* = 313)	Significance*^a^*
Measure				
Children with ARI (%)	48.6	29.7	69.6	*^,^**^,^***
No. of ARI episodes	238	114	496	*^,^**^,^***
Annual rate of ARI	3.49	1.63	6.89	*^,^**^,^***
Baseline characteristics (*n*)	294	303	313	
Age [years (mean ± SD)]	8.3 ± 1. 6	8.9 ± 0.8	8.3 ± 1	*^,^***
Females (%)	51.7	49.5	29.4	**^,^***
Weight [kg (mean ± SD)]	23.9 ± 5.5	27.7 ± 5.2	27.0 ± 5.9	*^,^**
Underweight children, WAZ < −2 SD (%)	4.4	2.3	3.2	
Height [cm (mean ± SD)]	120.4 ± 9.5	128.5 ± 7.6	126.4 ± 8.5	*^,^**^,^***
Stunted, HAZ < −2 SD (%)	28.2	9.6	8.6	*^,^**
BMI (mean ± SD)	16.4 ± 1.8	16.7 ± 2.4	16.8 ± 2.3	*^,^**
Survey response (*n*)	176	301	258	
Completed surveys (%)	60	99	76	*^,^**^,^***
Crowdedness (mean ± SD)	1.9 ± 1.1	1.2 ± 0.6	0.8 ± 0.4	*^,^**^,^***
Households with kerosene use (%)	4.1	1.7	2.9	
Households with firewood use (%)	18.1	1.3	0.5	*^,^**
Households with smokers (%)	25.5	30.5	17.6	***
Children with history of asthma (%)	1.1	3.6	2.1	
Blood tests (*n*)	99	90	106	
COHb [% (mean ± SD)]	0.70 ± 1.17	2.52 ± 1.12	5.09 ± 1.7	*^,^**^,^***
COHb > 2.5% (%)	1	43	92	*^,^**^,^***
Hematocrit [% (mean ± SD)]	41.6 ± 2.0	44.4 ± 2.4	43.8 ± 2.5	*^,^**

a Significance at *p* < 0.05:

* LT-school versus MT-school;

** LT-school versus HT-school;

*** MT-school versus HT-school.

**Table 3:** Average COHb concentrations in children attending LT-, MT-, and HT-schools and living in households with or without smokers, and with or without firewood/kerosene use.

	No.	COHb [% (mean ± SD)]	% COHb > 2.5%
LT-school			
Households with smokers	15	0.76 ± 0.59	0.00
Households without smokers	44	0.60 ± 0.29	2.27
MT-school			
Households with smokers	26	2.52 ± 1.1	42.3
Households without smokers	62	2.55 ± 1.25	41.93
HT-school			
Households with smokers	11	4.55 ± 1.75	90.9
Households without smokers	63	5.27 ± 1.62	93.6
LT-school			
Households with firewood/kerosene use	13	0.59 ± 0.26	0.00
Households without firewood/kerosene use	47	0.75 ± 0.58	2.21
MT-school			
Households with firewood/kerosene use	1	2.15	0.00
Households without firewood/kerosene use	86	2.49 ± 1.15	41.9
HT-school			
Households with firewood/kerosene use	1	3.24	100
Households without firewood/kerosene use	73	5.19 ± 1.64	93.2
